# A draft reference genome assembly of California Pipevine, *Aristolochia californica* Torr.

**DOI:** 10.1093/jhered/esae023

**Published:** 2024-04-15

**Authors:** Samridhi Chaturvedi, Merly Escalona, Mohan P A Marimuthu, Oanh Nguyen, Noravit Chumchim, Colin W Fairbairn, William Seligmann, Courtney Miller, H Bradley Shaffer, Noah K Whiteman

**Affiliations:** Department of Ecology and Evolutionary Biology, Tulane University, New Orleans, LA 70118, United States; Department of Integrative Biology, University of California, 142 Weill Hall #3200, Berkeley, United States; Department of Biomolecular Engineering, University of California Santa Cruz, Santa Cruz, CA 95064, United States; DNA Technologies and Expression Analysis Core Laboratory, Genome Center, University of California, Davis, CA 95616, United States; DNA Technologies and Expression Analysis Core Laboratory, Genome Center, University of California, Davis, CA 95616, United States; DNA Technologies and Expression Analysis Core Laboratory, Genome Center, University of California, Davis, CA 95616, United States; Department of Ecology and Evolutionary Biology, University of California, Santa Cruz, Santa Cruz, CA 95064, United States; Department of Ecology and Evolutionary Biology, University of California, Santa Cruz, Santa Cruz, CA 95064, United States; Department of Ecology and Evolutionary Biology, University of California, Los Angeles, CA 90095-7239, United States; Department of Ecology and Evolutionary Biology, University of California, Los Angeles, CA 90095-7239, United States; La Kretz Center for California Conservation Science, Institute of the Environment and Sustainability, University of California, Los Angeles, CA 90095-7239, United States; Department of Integrative Biology, University of California, 142 Weill Hall #3200, Berkeley, United States; Department of Molecular and Cell Biology, University of California, 142 Weill Hall #3200, Berkeley, CA 94720, United States

**Keywords:** angiosperm, Aristolochia, California Conservation Genomics Project, genomics

## Abstract

The California Pipevine, *Aristolochia californica* Torr., is the only endemic California species within the cosmopolitan birthwort family Aristolochiaceae. It occurs as an understory vine in riparian and chaparral areas and in forest edges and windrows. The geographic range of this plant species almost entirely overlaps with that of its major specialized herbivore, the California Pipevine Swallowtail Butterfly *Battus philenor hirsuta*. While this species pair is a useful, ecologically well-understood system to study co-evolution, until recently, genomic resources for both have been lacking. Here, we report a new, chromosome-level assembly of *A. californica* as part of the California Conservation Genomics Project (CCGP). Following the sequencing and assembly strategy of the CCGP, we used Pacific Biosciences HiFi long reads and Hi-C chromatin proximity sequencing technology to produce a de novo assembled genome. Our genome assembly, the first for any species in the genus, contains 531 scaffolds spanning 661 megabase (Mb) pairs, with a contig N50 of 6.53 Mb, a scaffold N50 of 42.2 Mb, and BUSCO complete score of 98%. In combination with the recently published *B. philenor hirsuta* reference genome assembly, the *A. californica* reference genome assembly will be a powerful tool for studying co-evolution in a rapidly changing California landscape.

## Introduction

**Fig. 1.**
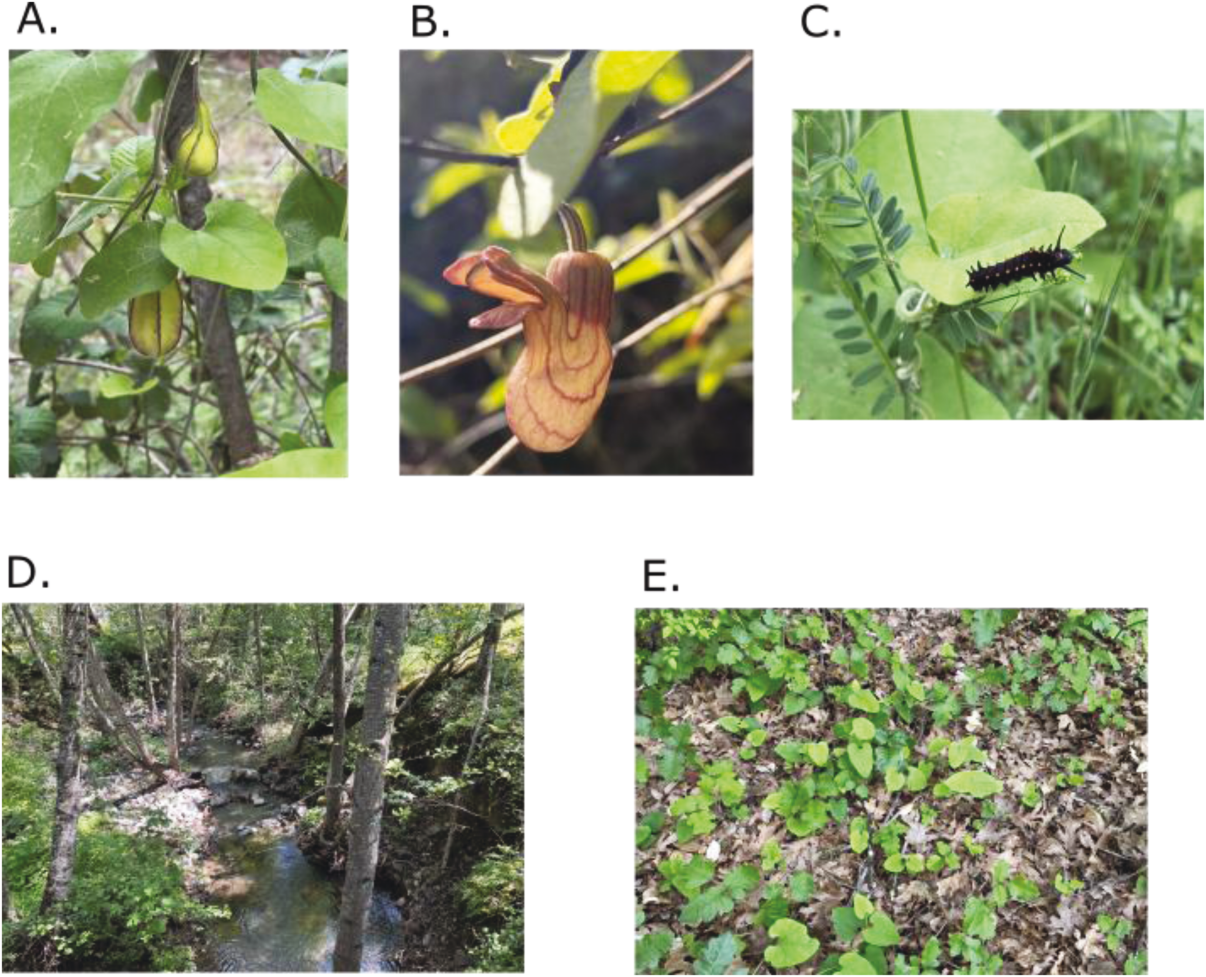
*Aristolochia californica* Torr. (California Pipevine) is a plant in the Aristolichaceae (birthwort) family endemic to north-central California in riparian streamside habitats in chaparral and the understory of redwood forests. A) *A. californica* vine and fruits, B) *A. californica* flower, C) *Battus philenor hirsuta* (California Pipevine Swallowtail Butterfly) caterpillar on *A. californica* leaf, D) Typical habitat of *A. californica* which occurs on the edges of rivers and streams, and E) *A. californica* vine spread across leaf bed. All photographs are copyright of S. Chaturvedi.

The cosmopolitan magnoliid “birthwort” family Aristolochiaceae contain ca. 550 tropical, sub-tropical, and temperate species, and most of these species (ca. 400 species) are members of the large genus *Aristolochia* (Kelly and González 2003; Neinhuis et al. 2004; Bliss et al. 2013). Birthwort species include lianas, shrubs, and tuberous herbs with unique floral morphologies primarily adapted for fly pollination (Kelly and González 2003). The California Pipevine, *Aristolochia californica* Torr., is the only endemic California species within the Aristolochiaceae (Ornduff et al. 2003; Levy and Connor 2004). In California, this species is a twining deciduous vine of the bay laurel-oak woodland understory in riparian areas, chaparral, and forest edges and windrows (Fig. 1D and E). The species range is restricted to north-central California, including the San Francisco Bay Area and the Great Central Valley, and almost entirely overlaps with that of its major specialized insect herbivore, the California Pipevine Swallowtail Butterfly *Battus philenor hirsuta* (Fordyce 2000). Riparian species are a critical element in our toolkit to manage and reduce the effect of wildfires in Northern California. Unfortunately, they are also highly susceptible to damage and range restriction, particularly in the human-modified stream and river ecosystems that characterize virtually all of California (Kobziar and McBride 2006). Largely a riparian species, *A. californica* is therefore highly susceptible to habitat degradation and destruction, and the species has been locally extirpated from several areas.

*Aristolochia californica* is also a useful system to study plant–insect interactions and co-evolution for several reasons. First, *A. californica* and congeners evolved a specialized floral morphology shaped as a pipe, leading to the vernacular “pipevine” or ‘Dutchman’s pipe’ (Fig. 1A and B). This pipe-like morphology, combined with specific floral features such as scents, nectaries, and trichomes, have facilitated the evolution of deceptive pollination systems in *Aristolochia* that include attraction, imprisonment, and release of specific pollinators (Fordyce and Agrawal 2001; Qin et al. 2021). Second, chemical analyses of *Aristolochia* species have revealed that these species naturally produce a class of nitrophenanthrene carboxylic acids known as aristolochic acids (AAs), which are highly toxic and carcinogenic in humans but are tolerated by some herbivores (Qin et al. 2021; Lin et al. 2022). *Aristolochia californica*, like its congeners, is the only host plant of *B. philenor hirsuta* (Fig. 1C). Evidence suggests that *B. philenor hirsuta* and other Troidinii butterflies use *Aristolochia* species as their only host plants and have evolved the ability to sequester AAs (Fordyce 2000; Silva-Brandão and Solferini 2007). Consistent with this unique feeding strategy, *A. californica* exhibits anti-predator defenses in response to herbivory from *B. philenor hirsuta* caterpillars by developing dense trichomes (Fordyce 2000; Fordyce and Agrawal 2001). Because it serves as the only food source for this iconic butterfly, the potential for co-evolution and co-extinction is particularly salient. Collectively, these features of *A. californica* make it a valuable model system to study plant evolution, co-evolution, insect-plant interactions, and medicinal plant chemistry.

Here, we report the first near-chromosome-level genome assembly for *A. californica*, sequenced and assembled as part of the California Conservation Genomics Project (CCGP). This genome assembly is the third whole genome assembly in this family (Li et al. 2019; Qin et al. 2021; Cui et al. 2022; Lin et al. 2022 for chloroplast genomes). Our sequencing approach generated an ~54-fold genome coverage based on its 592 Mb genome. The overarching goal of the CCGP is to discover patterns of genomic diversity across the state of California by sequencing the complete genomes of 153 carefully selected species (Shaffer et al. 2022). The ongoing efforts of the CCGP provide an unparalleled opportunity to use the reference genome sequences of both *B. philenor hirsuta* (Chaturvedi et al. 2023) and *A. californica*, combined with custom bioinformatic and landscape genomics analyses of both species (Chambers et al. 2023; Mirchandani et al. 2023), to gain a genome-level understanding of the demographic history, population structure, and co-evolution of both species. The CCGP data also affords the opportunity to identify genes that underlie pollination syndromes and medicinal properties of *A. californica*. This genome assembly fills an important phylogenetic gap in the CCGP program (Toffelmier et al. 2022) and will provide a foundational resource for future studies on the unique ecology, biogeography, evolutionary history, behavior, and conservation of this unique California endemic species.

## Methods

### Biological materials

Fresh buds, soft stems/petioles, and young leaves were collected from one individual by N.K.W. on April 10, 2021, from the University of California (UC), Berkeley Botanical Garden (Berkeley, CA; UCBG coordinates: 37.87118, −122.238632). Upon collection, the tissues were immediately stored under liquid nitrogen vapor in a dry shipper and then transferred to a -80 °C freezer for short-term storage. This plant was originally sampled by Roger Raiche (R.20769) of the UC Botanical Garden (Acc. number 82.1470) from an individual collected on October 19,1982 that is still growing in the garden. This individual was accessioned in the Jepson Herbarium at UC Berkeley on 25 October 1982. The location on the accession label is Butte County, CA, USA, North America. Further details on the label indicate that the sample was collected around the Feather River at 1,000 feet (ca. 305 m) above sea level (approximate coordinates: 39.589330976, −121.253832318). At this location, *A. californica* grows in shady, usually moist slopes in *Pinus ponderosa* or *Quercus* spp. forests. The collected tissues were divided into two tubes and shipped overnight on dry ice to the UC Davis Genome Center (Davis, CA) and UC Santa Cruz (Santa Cruz, CA) sequencing cores.

### Nucleic acid library preparation

High molecular weight (HMW) genomic DNA (gDNA) was extracted from young leaves (1.5 g) using the Nanobind Plant Nuclei Big DNA Kit as per the manufacturer’s instructions (Pacific Biosciences—PacBio, Menlo Park, CA) with the following modification. We used a nuclear isolation buffer supplemented with 350 mM sorbitol to resuspend ground tissue and during the first wash of the nuclei pellet. The extracted HMW DNA was further purified using the high-salt-phenol-chloroform method. The DNA purity was estimated using absorbance ratios (260/280 = 1.86 and 260/230 = 2.58) on a NanoDrop ND-1000 spectrophotometer. The final DNA yield (6 μg) was quantified using the Quantus Fluorometer (QuantiFluor ONE dsDNA Dye assay; Promega, Madison, WI). The size distribution of the HMW DNA was estimated using the Femto Pulse system (Agilent, Santa Clara, CA) and we found that 50% of the fragments were 125 kb or longer.

The HiFi SMRTbell library was constructed using the SMRTbell Express Template Prep Kit v2.0 (Pacific Biosciences, Menlo Park, CA; Cat. #100-938-900) according to the manufacturer’s instructions. HMW gDNA was sheared to a target DNA size distribution between 15 and 18 kb using Diagenode’s Megaruptor 3 system (Diagenode, Belgium; cat. B06010003). The sheared gDNA was concentrated using 0.45× of AMPure PB beads (Pacific Biosciences, Menlo Park, CA; Cat. #100-265-900) for the removal of single-strand overhangs at 37 °C for 15 min, followed by further enzymatic steps of DNA damage repair at 37 °C for 30 min, end repair and A-tailing at 20 °C for 10 min and 65 °C for 30 min, and ligation of overhang adapters v3 at 20 °C for 60 min. The SMRTbell library was purified and concentrated with 1× AMPure PB beads for nuclease treatment at 37 °C for 30 min and size selection using the PippinHT system (Sage Science, Beverly, MA; Cat #HPE7510) to collect fragments between 7 and 9 kb. The 15 to 20 kb average HiFi SMRTbell library was sequenced at UC Davis DNA Technologies Core (Davis, CA) using one 8M SMRT cell, Sequel II sequencing chemistry 2.0, and 30-hour movies each on a PacBio Sequel II sequencer.

The Omni-C library was prepared using the Dovetail^TM^ Omni-C^TM^ Kit (Dovetail Genomics, CA) according to the manufacturer’s protocol with slight modifications. First, specimen tissue (young leaves, ID: CCGP_79_NKW_82.1479) was thoroughly ground with a mortar and pestle under liquid nitrogen. Nuclear isolation was then performed using published methods (Workman et al. 2019). Subsequently, chromatin was fixed in place in the nucleus and digested under various conditions of DNase I until a suitable fragment length distribution of DNA molecules was obtained. Chromatin ends were repaired and ligated to a biotinylated bridge adapter followed by proximity ligation of adapter-containing ends. After proximity ligation, crosslinks were reversed, and the DNA was purified from proteins. Purified DNA was treated to remove biotin that was not internal to ligated fragments. An NGS library was generated using an NEB Ultra II DNA Library Prep kit (NEB, Ipswich, MA) with an Illumina-compatible y-adaptor. Biotin-containing fragments were then captured using streptavidin beads. The post-capture product was split into two replicates prior to PCR enrichment to preserve library complexity with each replicate receiving unique dual indices. The library was sequenced at the Vincent J. Coates Genomics Sequencing Lab (UC Berkeley; Berkeley, CA) on an Illumina NovaSeq 6000 platform (Illumina, CA) to generate approximately 100 million 2 × 150 bp read pairs per GB genome size.

### Nuclear genome assembly

We assembled the genome of *A. californica* following the CCGP assembly pipeline Version 5.0, as outlined in Table 1, which lists the tools and non-default parameters used in the assembly. First, we removed the remnants adapter sequences from the PacBio HiFi dataset using HiFiAdapterFilt (Sim et al. 2022) and generated the initial dual or partially phased diploid assembly (http://lh3.github.io/2021/10/10/introducing-dual-assembly) using HiFiasm (Cheng et al. 2021) on Hi-C mode, with the filtered PacBio HiFi reads and the Omni-C dataset. We then aligned the Omni-C data to both assemblies following the Arima Genomics Mapping Pipeline (https://github.com/ArimaGenomics/mapping_pipeline) and scaffolded both assemblies with SALSA (Ghurye et al. 2017, 2019).

**Table 1.** Assembly Pipeline and Software Used.

Assembly	Software and options^§^	Version
Filtering PacBio HiFi adapters	HiFiAdapterFilt	Commit 64d1c7b
K-mer counting	Meryl (*k* = 21)	1
Estimation of genome size and heterozygosity	GenomeScope	2
De novo *assembly (contiging)*	HiFiasm (Hi-C Mode, –primary, output p_ctg.hap1, p_ctg.hap2)	0.16.1-r375
Scaffolding		
Omni-C data alignment	Arima Genomics Mapping Pipeline	Commit 2e74ea4
Omni-C scaffolding	SALSA (-DNASE, -i 20, -p yes)	2
Gap closing	YAGCloser (-mins 2 -f 20 -mcc 2 -prt 0.25 -eft 0.2 -pld 0.2)	Commit 0e34c3b
Omni-C Contact map generation		
Short-read alignment	BWA-MEM (-5SP)	0.7.17-r1188
SAM/BAM processing	samtools	1.11
SAM/BAM filtering	pairtools	0.3.0
Pairs indexing	pairix	0.3.7
Matrix generation	cooler	0.8.10
Matrix balancing	hicExplorer (hicCorrectmatrix correct --filterThreshold -2 4)	3.6
Contact map visualization	HiGlass	2.1.11
	PretextMap	0.1.4
	PretextView	0.1.5
	PretextSnapshot	0.0.3
Manual curation tools	Rapid curation pipeline (Wellcome Trust Sanger Institute, Genome Reference Informatics Team)	Commit 4ddca450
Genome quality assessment		
Basic assembly metrics	QUAST (--est-ref-size)	5.0.2
Assembly completeness	BUSCO (-m geno, -l embryophyte_odb10)	5.0.0
	Merqury	2020-01-29
Contamination screening		
Local alignment tool	BLAST + (-db nt, -outfmt “6 qseqid staxids bitscore std,” -max_target_seqs 1, -max_hsps 1, -evalue 1e-25)	2.1
General contamination screening	BlobToolKit (PacBio HiFi Coverage, BUSCODB = embryophyta, NCBI Tax ID = 171875)	2.3.3

Software citations are listed in the text.

^§^Options detailed for non-default parameters.

Both haplotypes were manually curated by iteratively generating and analyzing their corresponding Omni-C contact maps. To generate the contact maps, we aligned the Omni-C data with BWA-MEM (Li 2013), identified ligation junctions, and generated Omni-C pairs using pairtools (Goloborodko et al. 2018). We generated a multi-resolution Omni-C matrix with cooler (Abdennur and Mirny 2020) and balanced it with hicExplorer (Ramírez et al. 2018). We used HiGlass (Kerpedjiev et al. 2018) and the PretextSuite (https://github.com/wtsi-hpag/PretextView; https://github.com/wtsi-hpag/PretextMap; https://github.com/wtsi-hpag/PretextSnapshot) to visualize the contact maps where we identified misassemblies and misjoins, and finally modified the assemblies using the Rapid Curation pipeline from the Wellcome Trust Sanger Institute, Genome Reference Informatics Team (https://gitlab.com/wtsi-grit/rapid-curation). Some of the remaining gaps (joins generated during scaffolding and curation) were closed using the PacBio HiFi reads and YAGCloser (https://github.com/merlyescalona/yagcloser). Finally, we checked for contamination using the BlobToolKit Framework (Challis et al. 2020).

### Genome quality assessment

We generated k-mer counts from the PacBio HiFi reads using Meryl (https://github.com/marbl/meryl). The k-mer counts were then used in GenomeScope2.0 (Ranallo-Benavidez et al. 2020) to estimate genome features including genome size, heterozygosity, and repeat content. To obtain general contiguity metrics, we ran QUAST (Gurevich et al. 2013). To evaluate genome quality and functional completeness we used BUSCO (Manni et al. 2021) with the Embryophyta ortholog database (embryophyta_odb10) which contains 1,614 genes. Assessment of base level accuracy (QV) and k-mer completeness was performed using the previously generated Meryl database and Merqury (Rhie et al. 2020). We further estimated genome assembly accuracy via BUSCO gene set frameshift analysis using the pipeline described in (Korlach et al. 2017).

Measurements of the size of the phased blocks is based on the size of the contigs generated by HiFiasm on HiC mode. We followed the quality metric nomenclature established by Rhie et al. (2021), with the genome quality code *x*.*y*.*P*.*Q*.*C*, where, *x* = log10[contig NG50]; *y* = log10[scaffold NG50]; *P* = log10 [phased block NG50]; *Q* = Phred base accuracy QV (quality value); *C* = % genome represented by the first “*n*” scaffolds, following a karyotype of 2*n* = 28 estimated from congeners (Genome on a Tree—GoaT; tax_tree (*A. californica*)). Quality metrics for the notation were calculated on the assembly for haplotype 1.

## Results

**Fig. 2.**
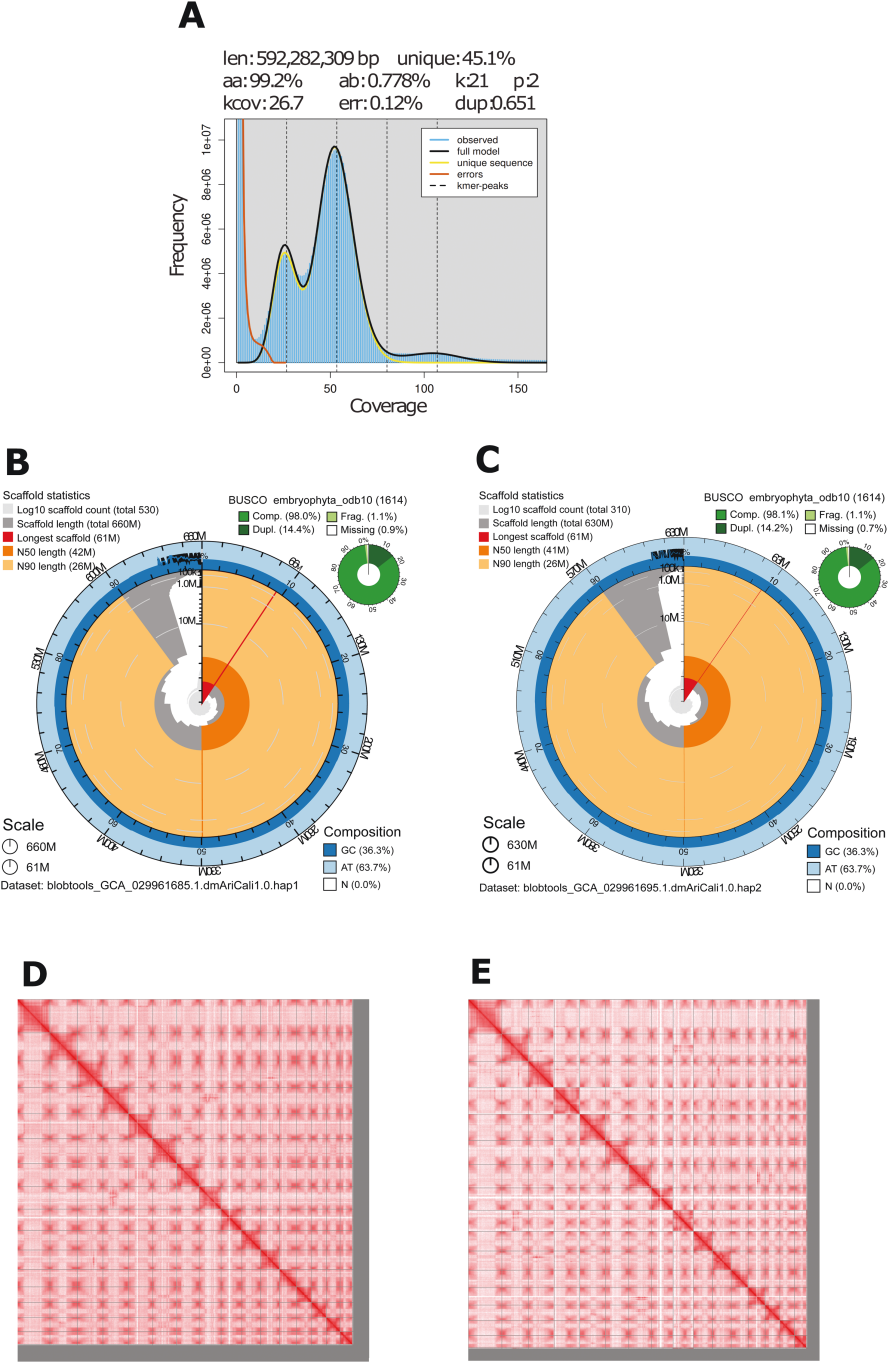
Visual overview of genome assembly metrics of *Aristolochia californica*. A) **K-mer spectra** output generated from PacBio HiFi data without adapters using GenomeScope2.0. The bimodal pattern observed corresponds to a diploid genome and the k-mer profile matches that of high (>1%) heterozygosity. K-mers covered at lower coverage and high frequency correspond to differences between haplotypes, whereas the higher coverage and lower frequency k-mers correspond to the similarities between haplotypes. **BlobToolKit Snail plot** showing a graphical representation of the quality metrics presented in Table 2 for the *A. californica* haplotype 1 (B) assembly and haplotype 2 (C) assembly. The plot circle represents the full size of the assembly. From the inside-out, the central plot covers length-related metrics. Line at 61M represents the size of the longest scaffold; all other scaffolds are arranged in size order moving clockwise around the plot and drawn in gray starting from the outside of the central plot. Dark and light orange arcs show the scaffold N50 and scaffold N90 values. The central light gray spiral shows the cumulative scaffold count with a white line at each order of magnitude. White regions in this area reflect the proportion of Ns in the assembly; the dark versus light blue area around it shows mean, maximum, and minimum GC vs. AT content at 0.1% intervals (Challis et al. 2020). **Hi-C Contact maps** for haplotype 1 (D) and haplotype 2 (E) genome assembly generated with PretextSnapshot. Hi-C contact maps translate the proximity of genomic regions in 3D space to contiguous linear organization. Each cell in the contact map corresponds to sequencing data supporting the linkage (or join) between two such regions.

The Omni-C and PacBio HiFi sequencing libraries generated 96.29 million read pairs and 2.44 million reads, respectively. The latter yielded ~54-fold coverage (N50 read length 13,759 bp; minimum read length 92 bp; mean read length 13,282 bp; maximum read length of 52,150 bp) based on the Genomescope 2.0 genome size estimation of 592.28 Mb. Based on PacBio HiFi reads, we estimated 0.12% sequencing error rate and 0.778% nucleotide heterozygosity rate. The k-mer spectrum based on PacBio HiFi reads (Fig. 2A) showed a bimodal distribution with two major peaks at ~26 and ~54-fold coverage, where peaks corresponded to homozygous and heterozygous states of a diploid species.

**Table 2.** Sequencing and assembly statistics, and accession numbers.

Bio Projects and Vouchers	CCGP NCBI BioProject			PRJNA720569			
	Genera NCBI BioProject			PRJNA766269			
	Species NCBI BioProject			PRJNA777140			
	NCBI BioSample			SAMN34142604			
	Specimen identification			CCGP_79_NKW_82.1479			
	NCBI genome accessions			Haplotype 1		Haplotype 2	
	Assembly accession			JARWKL000000000		JARWKM000000000	
	Genome sequences			GCA_029961685.1		GCA_029961695.1	
Genome Sequence	PacBio HiFi reads		Run	1 PACBIO_SMRT (Sequel II) run: 2.4M spots, 32.5G bases, 19.1Gb			
			Accession	SRX21227424			
	Omni-C Illumina reads		Run	2 ILLUMINA (Illumina NovaSeq 6000) runs: 96.3M spots, 29G bases, 9.7Gb			
			Accession	SRX21227425, SRX21227426			
Genome Assembly Quality Metrics	Assembly identifier (Quality code*)				dmAriCali1(6.7.P6.Q64.C96)		
	HiFi read coverage^§^			54.86X			
				Haplotype 1		Haplotype 2	
	Number of contigs			706		471	
	Contig N50 (bp)			6,530,752		6,370,861	
	Contig NG50^§^				7,384,122		7,006,831
	Longest contigs			15,797,741		21,344,178	
	Number of scaffolds			531		306	
	Scaffold N50			42,251,748		40,911,028	
	Scaffold NG50^§^				45,152,081		40,911,028
	Largest scaffold			61,303,474		60,509,716	
	Size of final assembly			661,636,370		633,851,113	
	Phased block NG50^§^				7,077,005		6,883,816
	Gaps per Gbp (# Gaps)			264(175)		260(165)	
	Indel QV (Frame shift)			47.15		48.13	
	Base pair QV			65.9682		65.9682	
				Full assembly = 64.8514			
	k-mer completeness			89.7974		89.0494	
				Full assembly = 98.9802			
	BUSCO completeness (embryophyta) *n* = 1614		*C*	*S*	*D*	*F*	*M*
		H1^‡^	98.00%	83.60%	14.40%	1.10%	0.90%
		H2^‡^	98.20%	84.00%	14.20%	1.10%	0.70%
	Organelles		# Partial/complete mitochondrial sequence				

*Assembly quality code *x*.*y*.*P*.*Q*.*C* derived notation, from Rhie et al. (2021). *x* = log10[contig NG50]; *y* = log10[scaffold NG50]; *P* = log10 [phased block NG50]; *Q* = Phred base accuracy QV (Quality value); *C* = % genome represented by the first “*n*” scaffolds, following a karyotype of 2*n* = 28 estimated from other species in the same genus. Quality code for all the assembly denoted by primary assembly (dmAriCali1.0.hap1).

^§^Read coverage and NGx statistics have been calculated based on the estimated genome size of 592.28 Mb.

^‡^(H1) Haplotype 1 and (H2) Haplotype 2 assembly values.

The final assembly (dmAriCali1) included two partially phased haplotypes that varied slightly in size compared to the estimated value from GenomeScope2.0 (Fig. 2A), as observed in other taxa (for example, see Pflug et al. 2020).

Haplotype 1 consists of 531 scaffolds spanning 661.61 Mb with contig N50 of 6.53 Mb, scaffold N50 of 42.25 Mb, largest contig of 15.79 Mb, and largest scaffold of 61.30 Mb. Haplotype 2 consists of 306 scaffolds, spanning 633.83 Mb with contig N50 of 6.37 Mb, scaffold N50 of 40.91 Mb, largest contig 21.34 Mb, and largest scaffold of 60.50 Mb. Assembly statistics are reported in Table 2, and graphical representations for the haplotype 1 and haplotype 2 assemblies are presented in Fig. 2B and C.

During manual curation, we generated a total of 32 breaks and 165 joins; 16 breaks were made per haplotype, with 87 joins on haplotype one, and 78 joins on haplotype two. We were able to close a total of 40 gaps, 21 on haplotype one and 19 on haplotype two. We did not remove any contigs due to contaminants.

Haplotype 1 has a BUSCO completeness score of 98.0% using the Embryophyta gene set, a per base quality (QV) of 63.99, a k-mer completeness of 89.79, and a frameshift indel QV of 47.15. Haplotype 2 has a BUSCO completeness score of 98.2% using the same gene set, a per base quality (QV) of 63.96, a k-mer completeness of 89.04, and a frameshift indel QV of 48.13. Both assemblies are highly contiguous based on Omni-C contact maps (Fig. 2D and E). We deposited both assemblies on NCBI (see Table 2 and Data Availability for details).

## Discussion

The genome assembly of the California Pipevine, *A. californica Torr.*, adds to the increasing abundance of genomic resources for plants in the family Aristolochiaceae and the magnoliid clade. This species is only the third species in the Aristolochiaceae for which a high-coverage whole genome has been sequenced and assembled (see Li et al. 2019; Lin et al. 2022 for chloroplast genomes), and the first for a North American member of the family. We used the recently available whole genome assemblies of *A. fimbriata* (Qin et al. 2021) and *A. contorta* (Cui et al. 2022) to provide comparative metrics to assess the quality of the genome assembly presented here.

Of the three species, the *A. californica* genome assembly has the highest contig N50 at 6.53 Mb (compared to *A. contorta* with a contig N50 of 2.63 Mb (Cui et al. 2022), and *A. fimbriata* with a genome size of 5 Mb (Qin et al. 2021)). Additionally, the *A. californica* assembly has the highest scaffold N50 values at 42.25 Mb, surpassing both *A. contorta* (30.38 Mb) and *A. fimbriata* (12.9 Mb) genomes. The *A. californica* genome assembly also has the highest BUSCO completeness scores, reaching 98%, while *A. contorta* and *A. fimbriata* genomes achieved scores of 90.28% (Cui et al. 2022) and 96.8% (Qin et al. 2021), respectively. Lastly, the *A. californica* assembly’s genome size is over twice as large, spanning 661.61 Mb (for haplotype 1) compared to the genome sizes for *A. contorta* (210 Mb) and *A. fimbriata* (300 Mb), respectively. Therefore, the CCGP sequencing strategy of using a combined sequencing approach of PacBio HiFi and Omni-C Illumina reads drastically improved the assembly quality and completeness as seen for the *A. californica* genome assembly.

Inter- and intra-specific genome size variation is prevalent in plants, particularly in angiosperms, where up to a 3-fold variation has been observed among closely related species (Landry and Aubin-Horth 2013; Dai et al. 2022). Genome size variation has also been documented among individuals of the same species (Boutte et al. 2020; Becher et al. 2021). This variation, whether at the interspecific or individual level, is primarily attributed to polyploidization resulting from autopolyploidy or allopolyploidy from past hybridization events (Dai et al. 2022), structural variation of large genomic regions (Boutte et al. 2020), or variation in transposition rates (Ågren and Wright 2011). Interestingly, *A. californica,* like its congeners, is likely a diploid species (Bliss et al. 2013). This is supported by our GenomeScope results which indicate two peaks typical of diploid genomes (Fig. 2A, Ranallo-Benavidez et al. 2020). The reason for genome size variation in the subgenus *Aristolochia* remains an open question which needs to be explored further.

The *A. californica* genome assembly is a crucial resource for advancing evolutionary research in angiosperms. Genomic resources for North American *Aristolochia* species are currently limited, and there is a lack of detailed understanding regarding their biogeographic and colonization history. Furthermore, *A. californica* is particularly intriguing in the context of plant–insect interactions, as *B. philenor hirsuta* butterflies exclusively complete their life cycle on plants of this species. Previous studies have identified phenotypic traits that confer resistance in *A. californica* and aristolochic acid tolerance in specialized caterpillars of *B. philenor hirsuta* (Fordyce 2000, 2001; Fordyce and Agrawal 2001), which could be the context for co-evolution dynamics between them. However, the lack of genomic resources for both species has limited our understanding of several aspects of their underlying evolutionary histories including potential interspecific co-evolution. This genome assembly combined with the recently published genome assembly of *B. philenor hirsuta* (Chaturvedi et al. 2023), another CCGP taxon, provides important resources to initiate genomic studies on co-evolution of this species pair. Also, aristolochic acids present in plants of *Aristolochia* genus are known to cause irreversible hepatotoxicity, nephrotoxicity, genotoxicity, and carcinogenicity (Zhang et al. 2022). This genome assembly can be useful in comparative genomic analysis to identify the biosynthetic pathways associated with aristolochic acid generation to study medicinal biochemistry for human benefit.

Finally, the current geographic range of this species overlaps with regions that are highly susceptible to wildfires and the effects of climate change. This genome reference fills an important phylogenetic gap in our current collection of reference genomes of California endemics (Toffelmier et al. 2022), and will facilitate future studies on the genomic basis of adaptation to a rapidly changing climate (Fiedler et al. 2022), predicting vulnerability of this species to extinction, and on plant-herbivore co-evolution.

## Data Availability

Data generated for this study are available under NCBI BioProject PRJNA777140. Raw sequencing data for sample CCGP_79_NKW_82.1479 (UC Botanical Garden Accession number 82.1479; NCBI BioSample SAMN34142604) are deposited in the NCBI Short Read Archive (SRA) under SRR25496339 for PacBio HiFi sequencing data, and SRR25496337 and SRR25496338 for the Omni-C Illumina sequencing data. GenBank accessions for both haplotypes are GCA_029961685.1 (dmAriCali1.0.hap1) and GCA_029961695.1 (dmAriCali1.0.hap2); and for genome sequences JARWKL000000000 and JARWKM000000000. Assembly scripts and other data for the analyses presented can be found at the following GitHub repository: www.github.com/ccgproject/ccgp_assembly.
